# Deceased-Donor Apolipoprotein L1 Renal-Risk Variants Have Minimal Effects on Liver Transplant Outcomes

**DOI:** 10.1371/journal.pone.0152775

**Published:** 2016-04-07

**Authors:** Casey R. Dorr, Barry I. Freedman, Pamela J. Hicks, W. Mark Brown, Gregory B. Russell, Bruce A. Julian, Stephen O. Pastan, Michael D. Gautreaux, Amutha Muthusamy, Srinath Chinnakotla, Vera Hauptfeld, Robert A. Bray, Allan D. Kirk, Jasmin Divers, Ajay K. Israni

**Affiliations:** 1 Minneapolis Medical Research Foundation, Minneapolis, Minnesota, United States of America; 2 Department of Medicine, Hennepin County Medical Center, University of Minnesota, Minneapolis, Minnesota, United States of America; 3 Department of Internal Medicine, Section on Nephrology, Wake Forest School of Medicine, Winston-Salem, North Carolina, United States of America; 4 Center for Genomics & Personalized Medicine Research, Wake Forest School of Medicine, Winston-Salem, North Carolina, United States of America; 5 Department of Biostatistics, Wake Forest School of Medicine, Winston-Salem, North Carolina, United States of America; 6 Department of Medicine, Division of Nephrology, University of Alabama at Birmingham School of Medicine, Birmingham, Alabama, United States of America; 7 Department of Medicine, Renal Division, Emory University School of Medicine, Atlanta, Georgia, United States of America; 8 General Surgery & HLA Immunogenetics Lab, Wake Forest School of Medicine, Winston-Salem, North Carolina, United States of America; 9 Department of Surgery, University of Minnesota, Minneapolis, Minnesota, United States of America; 10 Alabama Regional Histocompatibility Laboratory at UAB, University of Alabama at Birmingham School of Medicine, Birmingham, Alabama, United States of America; 11 Department of Pathology & Lab Medicine, Emory School of Medicine, Atlanta, Georgia, United States of America; 12 Department of Surgery, Duke University School of Medicine, Durham, North Carolina, United States of America; 13 Department of Epidemiology and Community Health, University of Minnesota School of Medicine, Minneapolis, Minnesota, United States of America; University of Toledo, UNITED STATES

## Abstract

**Background:**

Apolipoprotein L1 gene (*APOL1*) G1 and G2 renal-risk variants, common in populations with recent African ancestry, are strongly associated with non-diabetic nephropathy, end-stage kidney disease, and shorter allograft survival in deceased-donor kidneys (autosomal recessive inheritance). Circulating APOL1 protein is synthesized primarily in the liver and hydrodynamic gene delivery of *APOL1* G1 and G2 risk variants has caused hepatic necrosis in a murine model.

**Methods:**

To evaluate the impact of these variants in liver transplantation, this multicenter study investigated the association of *APOL1* G1 and G2 alleles in deceased African American liver donors with allograft survival. Transplant recipients were followed for liver allograft survival using data from the Scientific Registry of Transplant Recipients.

**Results:**

Of the 639 liver donors evaluated, 247 had no *APOL1* risk allele, 300 had 1 risk allele, and 92 had 2 risk alleles. Graft failure assessed at 15 days, 6 months, 1 year and total was not significantly associated with donor *APOL1* genotype (p-values = 0.25, 0.19, 0.67 and 0.89, respectively).

**Conclusions:**

In contrast to kidney transplantation, deceased-donor *APOL1* G1 and G2 risk variants do not significantly impact outcomes in liver transplantation.

## Introduction

Two coding variants in the apolipoprotein L1 gene (*APOL1*) are associated with non-diabetic nephropathy [1] and end-stage kidney disease [2] in African Americans [1,2,3,4]. Strong association of these *APOL1* renal-risk variants is also seen with chronic kidney disease in African populations [5]. These *APOL1* risk variants include a G1 risk allele with two single-nucleotide polymorphisms (SNPs) (rs73885319; rs60910145) and the G2 risk allele which is an insertion/deletion (rs71785313). Approximately 13% of African Americans carry two *APOL1* renal-risk alleles [6], placing them at high risk for subsequent kidney disease. These alleles also predispose to lower levels of calcified atherosclerotic plaque, although effects on cardiovascular disease remain controversial [7,8]. It is not known how the *APOL1* G1 and G2 variants lead to kidney disease. *APOL1* mRNA is widely distributed and expressed in many tissues, including liver [9], and can be found circulating in plasma [10]. APOL1 protein is a component of high-density lipoprotein (HDL) cholesterol and is protective against infection with trypanosomes [11,12]. APOL1 has also been identified as a plasma biomarker for HCV-induced liver fibrosis [13] and variants are cytotoxic in rat hepatocytes, in part, regulated by autophagy [14].

Recently, *APOL1* high-risk genotypes were reported to induce liver injury in murine models [12] which has led us to hypothesize that these genotypes may also induce liver injury in humans and thus might impact outcomes after liver transplantation. In a murine model, the G1 *APOL1* variant caused more severe hepatic necrosis than did the G2 variant [12]. Specifically, mice were treated with hydrodynamic gene delivery of G1 or G2 *APOL1* variants, which led to low levels of APOL1 G1 in the sera [12], but the *APOL1* G1 variant was primarily found in the liver and produced severe hepatic necrosis [12]. Although less severe than *APOL1* G1, the G2 variant also caused hepatic necrosis in the mouse model [12]. Because *APOL1* G1 and G2 variants caused hepatic necrosis in murine models and are found in high frequency in African Americans, we investigated the role of the *APOL1* G1 and G2 genotypes in deceased African American liver donors on the clinical outcomes in liver-transplant recipients. We sought to determine whether donor *APOL1* renal-risk alleles were associated with allograft survival after liver transplantation.

## Methods

### Deceased-Donor Samples and Liver-Transplant Recipient Outcomes

DNA samples were received from the Organ Procurement Organizations (OPOs) in Genomics of Deterioration of Kidney Allograft Function study (DeKAF Genomics) [15], and the Emory University, Wake Forest, and University of Alabama at Birmingham Schools of Medicine. DNA extracted from blood, lymph nodes or spleen from deceased African American liver donors at these centers and aliquots of DNA from DeKAF Genomics were sent to Wake Forest School of Medicine for *APOL1* renal-risk variant genotyping. The DeKAF Genomics study OPOs included LifeSource (Minnesota), LifeQuest (Florida), New Jersey Organ & Tissue Sharing Network, Organ Donor Center of Hawaii, Southwest Transplant Alliance (Texas), One Legacy (California), New England Organ Bank (Massachusetts), LifeBanc (Ohio), and Louisiana Organ Procurement Agency.

The analysis of donors for livers recovered and/or transplanted at DeKAF Genomics, Emory University, Wake Forest, and University of Alabama at Birmingham resulted in a total of 639 liver transplantations performed at 78 centers between April 19, 1998 and August 26, 2013. Deceased-donor DNA samples were identified by United Network of Organ Sharing (UNOS) identification numbers. This study used clinical data from the Scientific Registry of Transplant Recipients (SRTR) to assess outcomes [16,17]. Wake Forest School of Medicine received Institutional Review Board (IRB) approval for genotyping DNA samples and linking outcomes to liver-transplant recipients based on UNOS identification numbers in SRTR. The SRTR data system includes data on all donors, wait-listed candidates, and transplant recipients in the United States, as submitted by the members of the Organ Procurement and Transplantation Network (OPTN) [16,17]. The Health Resources and Services Administration in the United States Department of Health and Human Services provided oversight to the activities of the OPTN and SRTR contractors. The clinical and research activities reported are consistent with the Principles of the Declaration of Istanbul as outlined in the Declaration of Istanbul on Organ Trafficking and Transplant Tourism.

The next of kin of deceased donors gave a written, general consent for research. The study received samples for DNA extraction only for those deceased donors with this consent for research. The deceased donor genotype was linked with the SRTR using the donor identification number and no recipient identifiers were available to the study. The IRBs at Hennepin County Medical Center and Wake Forrest University approved this consent process. None of the transplant donors were from a vulnerable population and all donors or next of kin provided written informed consent that was freely given.

### Genotyping

Two SNPs in the *APOL1* G1 risk allele (rs73885319; rs60910145) and an insertion/deletion for the G2 risk allele (rs143830837) were genotyped using a custom assay designed at Wake Forest on the Sequenom platform (San Diego, California). Genotype calls were visually inspected for quality control (3,14). Genotyping of 15 blind duplicates resulted in 100% concordance rate genotyping efficiency for the three loci.

*APOL1* genotype defined as 0 refers to deceased donors who did not carry a risk allele at either G1 or G2. *APOL1* genotype defined as 1 refers to deceased donors who were heterozygous at either G1 or G2. The *APOL1* genotype defined as 2 refers to deceased donors who carried 2 risk variants at either G1, G2, or were compound heterozygotes (one variant at G1 and one variant at G2). Thus, *APOL1* genotypes of 0, 1, or 2 indicate 0, 1, or 2 copies of G1 or G2 alleles, respectively.

### Statistical Analysis

The distribution of demographic variables for recipients and deceased liver donors, based on donor *APOL1*-risk genotypes, was contrasted using Kruskal-Wallis tests (continuous variables) and chi-square tests (categorical variables). The Kruskal-Wallis test is robust to deviations from normality. The main outcome was time to liver allograft failure, determined by the interval between the date of transplantation and the date of allograft loss prior to November 30, 2013. In those with a functioning allograft, the final observation date was censored in the event of death with function or at most recent follow-up prior to the censoring date. Cox proportional hazard models were developed. Missing genotype and phenotype data were excluded. The variables considered in this analysis had low counts of missing data (<5%), limiting the appeal for data imputation techniques. A p-value of < 0.05 was considered to be statistically significant.

## Results

Table 1 shows the donor and recipient demographic data, based on the *APOL1* genotype of the African American deceased donors. In total, there were 639 African American deceased liver donors, of whom 247 had 0 *APOL1* renal-risk alleles, 300 had 1 *APOL1* renal-risk allele, and 92 had 2 *APOL1* renal-risk alleles. Although all donors were African American; only 103 liver transplant recipients (16.1%) were African American. For the donors who had 0, 1, or 2 *APOL1* risk alleles, there was no significant difference in the demographic characteristics of the donors or recipients (Table 1).

**Table 1:** Demographic data for liver transplant recipients, based on *APOL1* genotype of African American deceased donors.

	APOL1* *renal-risk variant number in liver donors*			
Variable	*APOL1* = 0 (N = 247)	*APOL1* = 1 (N = 300)	*APOL1* = 2 (N = 92)	P-value
Donor age (years)	36.3 (17.5) 37	37.9 (17.7) 41	37 (15.4) 40	0.64
Recipient age at transplant (years)	49.8 (16.5) 54	50.1 (16.4) 55	52.8 (14) 54	0.57
Recipient body mass index (kg/m^2^)	27.4 (6.3) 26.9	27.9 (6.6) 27.5	28.6 (5.9) 28.6	0.13
Cold ischemia time (hours)	6.7 (3) 6.3	7.1 (3.7) 6.6	6.9 (3.3) 6.2	0.46
Recipient gender (male)	170 (68.8%)	211 (70.3%)	64 (69.6%)	0.93
Donor gender (male)	141 (57.1%)	171 (57%)	50 (54.3%)	0.89
Recipient ethnicity (African American)	38 (15.4%)	50 (16.7%)	15 (16.3%)	0.92
Standard-criteria donor (Yes)	54 (22.3%)	78 (26.4%)	14 (15.9%)	0.11
Non-heart-beating donor (Yes)	5 (2%)	3 (1%)	1 (1.1%)	0.57
Recipient drug induction (Yes)	141 (58.8%)	183 (63.1%)	65 (70.7%)	0.13
Previous transplant—liver (Yes)	17 (6.9%)	20 (6.7%)	11 (12%)	0.22
Recipient previous hepatic malignancy (Yes)	2 (0.8%)	6 (2%)	3 (3.3%)	0.27
**Recipient HCV Status**				0.76
Negative (<30 inhibition %)	141 (57.1%)	151 (50.5%)	48 (52.7%)	
Unknown (30 ≤ U < 70 inhibition %)	5 (2.0%)	6 (2.0%)	1 (1.1%)	
Positive (≥ 70 inhibition %)	89 (36.0%)	123 (41.1%)	35 (38.5%)	
Not Done	12 (4.9%)	19 (6.4%)	7 (7.7%)	
**Recipient primary diagnosis**				0.91
Acute hepatic necrosis	8 (3.2%)	151 (50.5%)	4 (4.3%)	
HCV	70 (28.3%)	79 (26.3%)	26 (28.3%)	
Alcoholic liver disease	35 (14.2%)	57 (19.0%)	19 (20.7%)	
Cholestatic disease	26 (10.5%)	27 (9.0%)	6 (6.5%)	
Malignancy	23 (9.3%)	31 (10.3%)	9 (9.8%)	
Other	85 (34.4%)	95 (31.7%)	28 (30.4%)	

* *APOL1* genotype defined as 0 refers to deceased donors who did not carry either G1 or G2 risk allele. *APOL1* genotype defined as 1 refers to deceased donors who were heterozygous at either G1 or G2. The *APOL1* genotype defined as 2 refers to deceased donors who carried 2 risk variants at either G1, G2, or were compound heterozygotes (one G1 variant and one G2 variant). Thus, *APOL1* genotypes of 0, 1, or 2 indicate 0, 1, or 2 copies of G1 or G2 alleles, respectively. Data are shown as [mean (standard deviation) median], or as frequency with percentage of total, unless otherwise stated.

Table 2 shows the allograft survival outcomes for liver recipients, based on the *APOL1* genotype of the deceased donor. Graft failure at 15 days, 6 months, 1 year, and total was not associated with *APOL1* genotype, p-values = 0.25, 0.19, 0.67, and 0.89, respectively. The median (standard deviation) follow-up time was 41.5 (31.1) months. Transplant recipients receiving a liver from donors with 2 *APOL1* risk alleles died 21.7% of the time, compared to 27.1% with 1 risk allele or 32.7% with 0 risk alleles. These data demonstrate that transplantation of livers from deceased donors with 0, 1, or 2 *APOL1* risk alleles was not associated with allograft outcome.

**Table 2:** Allograft function outcomes for liver transplant recipients, based on *APOL1* genotype of African American deceased donors.

	*APOL1* renal-risk variant number in liver donors			
Variable	*APOL1* = 0 (N = 247)	*APOL1* = 1 (N = 300)	*APOL1* = 2 (N = 92)	P-value
Allograft failure within 15 days (Yes)	3 (1.2%)	7 (2.3%)	0 (0.0%)	0.25
Allograft failure within 6 months (Yes)	8 (3.2%)	15 (5.0%)	1 (1.1%)	0.19
Allograft failure within 1 year (Yes)	11 (4.5%)	18 (6%)	4 (4.3%)	0.67
Allograft failure, total (Yes)	20 (8.1%)	23 (7.7%)	6 (6.5%)	0.89
Recipient death (Yes)	67 (27.1%)	98 (32.7%)	20 (21.7%)	0.09
Death with functioning allograft (Yes)	53 (21.5%)	72 (24%)	12 (13%)	0.08
Allograft survival (months)*	39.9 (30.1) 36	40.4 (31.1) 36	49.1 (33.5) 48	0.07

* The numbers are: mean (standard deviation) and median.

**Fig 1:**
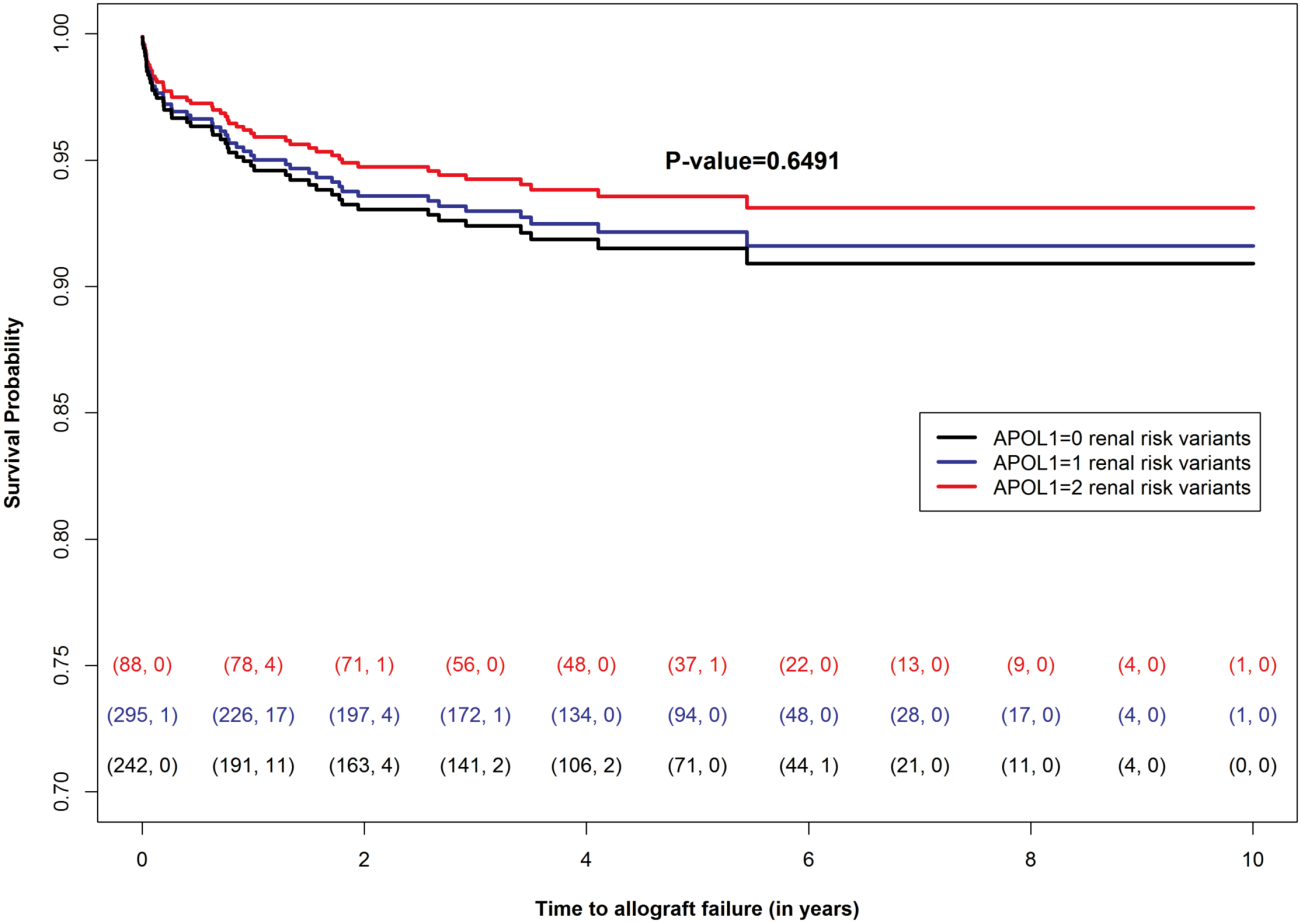
Allograft Survival Time with 0, 1, or 2 *APOL1* Renal Risk Variants in Donor Liver. The number of censored events is the difference between the number of transplantations that started the year and the number of events observed during the year. These numbers are shown in parentheses at the bottom of the plot; each color corresponds to a specific *APOL1* risk group. With a P-value of 0.6491 there is little correlation between liver allograft survival and the *APOL1* renal risk variants. P-value is from the fully adjusted Cox proportional model.

Fig 1 shows the liver allograft survival probability over time dependent on having 0, 1, or 2 of the *APOL1* renal-risk variants. The P-value of 0.6491 indicates the number of the *APOL1* renal-risk variants were not associated with liver allograft survival.

Table 3 shows the multivariate model which indicated that *APOL1* genotype was not associated with allograft survival, using either a recessive or an additive model. An effect of recipient age on liver allograft failure was detected.

**Table 3:** Multivariate association with liver allograft failure based on deceased donor *APOL1* genotype. Recessive and additive models are in the table. Both models use the full data set of *APOL1* genotyped liver donors (N = 639) in analysis and 49 events.

Variable	Hazard Ratio	95% Confidence Interval	P-value
**Recessive Model**			
*APOL1*	0.80	(0.35,1.86)	0.61
Recipient gender (female)	0.79	(0.43,1.47)	0.49
Recipient ethnicity (African American)	0.59	(0.25,1.41)	0.24
Recipient age at transplant	0.98	(0.96,0.99)	0.01
Donor age	1.01	(0.98,1.04)	0.35
Expanded-criteria donor (No)	0.71	(0.26,1.90)	0.49
Previous liver transplant	1.84	(0.73,4.65)	0.20
Recipient previous hepatic malignancy	1.35	(0.15,11.87)	0.79
**Recipient HCV Status** (Ref = N)			0.38
Not Detected	2.33	(0.83,6.49)	0.11
Positive	1.32	(0.68,2.56)	0.41
Unknown	1.89	(0.31,11.64)	0.49
**Donor source** (Ref = Wake Forest)			0.45
Emory University	0.84	(0.42,1.65)	0.61
DeKAF Genomics	0.56	(0.25,1.27)	0.17
University of Alabama at Birmingham	0.53	(0.18,1.57)	0.25
**Additive Model**			
*APOL1*	0.91	(0.61,1.37)	0.65
Recipient gender (female)	0.79	(0.43,1.48)	0.47
Recipient ethnicity (African American)	0.59	(0.25,1.41)	0.24
Recipient age at transplant	0.98	(0.96,0.99)	0.01
Donor age	1.01	(0.98,1.04)	0.36
Expanded-criteria donor (No)	0.69	(0.26,1.86)	0.47
Previous liver transplant	1.81	(0.72,4.58)	0.21
Recipient previous hepatic malignancy	1.35	(0.15,11.99)	0.79
**Recipient HCV Status** (Ref = N)			0.38
Not Detected	2.33	(0.84,6.45)	0.10
Positive	1.33	(0.69,2.57)	0.40
Unkown	1.88	(0.3,11.80)	0.50
**Donor source** (Ref = Wake Forest)			0.46
Emory University	0.84	(0.43,1.66)	0.62
DeKAF Genomics	0.57	(0.25,1.27)	0.17
University of Alabama at Birmingham	0.54	(0.18,1.58)	0.26

## Discussion

*APOL1* G1 and G2 risk alleles are common in African Americans and markedly influence susceptibility to non-diabetic kidney disease; furthermore, the two renal-risk-variant genotypes in deceased donors accelerate renal-allograft failure. This study is the first to examine liver transplantation outcomes based on deceased-donor *APOL1* renal-risk allele genotypes. Outcomes for 639 liver transplants from African American deceased donors revealed that donor *APOL1* G1 and G2 risk alleles did not significantly associate with liver allograft survival or rates of liver allograft rejection.

We hypothesized that *APOL1* risk alleles in donors had the potential to influence outcomes of liver transplantation because two *APOL1* G1 and/or G2 risk alleles in deceased donors are associated with shorter renal allograft survival [1]. Circulating APOL1 is predominantly synthesized in the liver [14,18]. Furthermore, hydrodynamic gene delivery of *APOL1* G1 and G2 variants caused liver necrosis in mice [12], and *APOL1* is a biomarker for HCV-induced liver fibrosis [13]. *APOL1* mRNA is highly expressed in the liver [9]; the Protein Atlas shows that at least 100 fragments per kilobase of transcript per million fragments mapped (FPKM) of *APOL1* are expressed in liver. *APOL1* was also expressed in liver based on analysis of tissue by western blot using multiple primary antibodies and by qRT-PCR [19]. It has also been reported that overexpressed *APOL1* G0 (without G1 or G2 allele) causes cell death in multiple human cell lines, DLD-1 (colorectal cancer), HepG2 (liver cancer), MCF-7 (breast cancer), and LNCaP, PC3, and DU145 (prostate cancer) [20]. The predominant mechanism of cytoxicity is autophagy [20]. Moreover, it has been determined that the G1 and G2 risk alleles are cytotoxic in hepatocytes, in part, regulated by autophagy [14]. Our study aimed to determine if the *APOL1* alleles in deceased donors influence outcomes in human liver transplant recipients. Although *APOL1* RNA and protein are present in liver tissue [14,18], our study determined that *APOL1* G1 and/or G2 variants in donor liver do not associate with liver allograft survival.

Our study is innovative because it enlisted multiple transplant centers and linked outcomes with the SRTR. SRTR uniformly collects data on all patient deaths and allograft failures leading to relisting. Nonetheless, this report has limitations. Differences in practice patterns at each transplant center were not taken into account; however, adjustment was performed for the source of the donor samples. With 49 liver failures observed in the 639 transplantations we have limits on our statistical power. However, this analysis suggests that the effect size of *APOL1* G1 and G2 alleles on liver allograft failure is likely to be small with a hazard ratio of 0.8 (or 1.25 for a positive- association effect) under a recessive mode of inheritance. Furthermore, another limitation is that the donors were self-identified as African American. Although participant DNA samples in DeKAF Genomics have not yet been screened for African ancestry proportion, samples from Wake Forest, Emory and the University of Alabama at Birmingham underwent a genome-wide association study and all had between 19% and 96% African ancestry across the genome, confirming self-reported ethnicity. Thirteen percent of the general African American population has two *APOL1* renal-risk variants and 39% have one; these variants appear to have originated in sub-Saharan Africa and are virtually limited to populations with recent African ancestry. Among the 639 liver donors in this report, 61.3% (392 of 639) had one or two *APOL1* renal-risk variants, further supporting the accuracy of the self-reported ethnicities. Another limitation is that we did not track if any of these deceased liver transplant donors provided kidneys for transplantation as well or the clinical outcomes of such kidney recipients. However, a previous study determined that G1 and G2 *APOL1* alleles in deceased kidney donors were associated with shorter renal allograft survivals [21,22]. Additionally, we could not assess mild degrees of liver dysfunction based on biopsy data because this information is lacking in SRTR. While there are limitations to this study, such limitations are common when studying effects of human genetic factors on clinical outcomes.

In summary, although donor *APOL1* renal-risk alleles have been associated with shorter renal allograft survival after deceased donor kidney transplantation, they are not associated with adverse outcomes after liver transplantation. There appears to be no benefit of genotyping *APOL1* G1 or G2 alleles in deceased African Americans donors to improve outcomes after liver transplantation. In contrast to kidney donation, if a liver donor has high-risk G1 and G2 *APOL1* alleles, the alleles do not necessarily confer high-risk for liver allograft failure.
